# A cost‐effective DNA extraction protocol for long‐read sequencing in non‐model plants

**DOI:** 10.1002/aps3.70055

**Published:** 2026-05-07

**Authors:** Sofia Gaischuk, Cintia F. Marchetti, Ezequiel Petrillo, Fernando A. Rabanal, Detlef Weigel, Nicolás Bellora, M. Verónica Arana

**Affiliations:** ^1^ Instituto de Investigaciones Forestales y Agropecuarias Bariloche, Instituto Nacional de Tecnología Agropecuaria, Estación Experimental Bariloche – Consejo Nacional de Investigaciones Científicas y Técnicas (INTA EEA Bariloche‐CONICET) San Carlos de Bariloche R8403DVZ Río Negro Argentina; ^2^ Instituto de Fisiología, Biología Molecular y Neurociencias, CONICET–Universidad de Buenos Aires Buenos Aires C1428EHA Argentina; ^3^ Department of Molecular Biology Max Planck Institute for Biology Tübingen Tübingen 72076 Germany; ^4^ Institute for Bioinformatics and Medical Informatics University of Tübingen Tübingen 72076 Germany; ^5^ Laboratorio de Genómica Computacional, Instituto de Tecnologías Nucleares para la Salud, Consejo Nacional de Investigaciones Científicas y Técnicas San Carlos de Bariloche 19 8400 Argentina

**Keywords:** DNA extraction, high‐molecular‐weight DNA, non‐model tree species, plant genomics, third‐generation sequencing

## Abstract

**Premise:**

Third‐generation sequencing has revolutionized genomics, enabling in‐depth analysis of genome sequence, structure, and epigenetic features. Yet, extracting high‐quality DNA for long‐read sequencing remains a bottleneck—particularly in non‐model plants, such as mature trees growing in natural environments, which often contain abundant endogenous compounds that hinder extraction and downstream applications.

**Methods and Results:**

We developed an optimized, robust, and cost‐effective DNA extraction protocol that yields high‐quality DNA suitable for Oxford Nanopore Technologies and PacBio sequencing. Validation across diverse taxa—including six *Nothofagus* species, gymnosperms endemic to Andean–Patagonian forests, exotic conifers of commercial value, and model plants—demonstrated consistently high DNA purity (A_260_/A_280_ > 1.8, A_260_/A_230_ > 2.0) and fragment sizes ≥30 kbp. Downstream sequencing confirmed suitability for applications requiring long, intact molecules and base modification detection.

**Conclusions:**

Compared to commercial kits and standard protocols, this approach achieved superior DNA integrity and yield without specialized equipment, offering an accessible solution for researchers working with challenging plant species.

Next‐generation sequencing (NGS) platforms provide high‐throughput, cost‐effective access to genomic information, even for non‐model species (Das and Chen, 2023). These technologies have enabled extensive research, allowing scientists to explore genetic information in ways that were not possible before, and have become essential tools across diverse scientific disciplines (Satam et al., 2023). This progress has significantly impacted global plant genomic research by providing accurate data generation critical for studying plant biology and crucial aspects of plant life, such as adaptation to diverse environments (Kwon et al., 2024).

Among NGS approaches, third‐generation sequencing technologies represent a cutting‐edge leap in DNA sequencing, particularly due to their ability to perform long‐read sequencing (Satam et al., 2023). These methods are particularly promising for overcoming challenges associated with sequencing and assembling large, repetitive, and complex genomes, such as plant genomes (Koo et al., 2023). Single‐molecule real‐time (SMRT) sequencing from Pacific Biosciences (PacBio) and nanopore sequencing from Oxford Nanopore Technologies (ONT) are the most common third‐generation sequencing technologies. Although these technologies offer advantages over short‐read technologies, DNA sample preparation remains challenging due to the requirement for high‐quality, ultra‐pure, and undamaged DNA, as both its quality and fragment size directly impact sequencing results. Difficulties are greatly exacerbated in plant species exhibiting complex tissue biochemistry, characterized by high levels of polysaccharides, phenolic compounds, and secondary metabolites. These compounds can damage DNA or inhibit enzyme reactions needed in downstream applications, such as library preparation (Lutz et al., 2011; Aboul‐Maaty and Oraby, 2019; Paajanen et al., 2019). Therefore, developing simple and efficient methods for isolating high‐molecular‐weight (HMW) DNA from biochemically challenging plant species suitable for third‐generation sequencing is essential (Kang et al., 2023).

Here, we present a DNA isolation method optimized for non‐model tree species growing in wild environments. The protocol begins with a nuclei purification step to eliminate most cellular components, followed by cetyltrimethylammonium bromide (CTAB)‐based nuclei lysis. DNA is then cleaned and precipitated using organic solvents and alcohol, and further purified using magnetic beads. This protocol yields high‐quality, high‐quantity, HMW DNA that was successfully sequenced using both PacBio and ONT platforms. It is cost‐effective and suitable for laboratories with basic equipment and limited resources. The method proved effective for extracting HMW DNA from all plant species tested, including six *Nothofagus* Blume species and three gymnosperms endemic to the Andean–Patagonian forests, two commercially relevant gymnosperm species, as well as the model plants *Populus trichocarpa* Torr. & A. Gray and *Arabidopsis thaliana* (L.) Heynh. We present quantification data, capillary electrophoretic profiles obtained by a Fragment Analyzer 5200 system (Agilent Technologies, Santa Clara, California, USA), and examples of ONT and PacBio sequencing metrics that demonstrate the efficacy of the method in obtaining DNA of the required quality for third‐generation sequencing.

## METHODS AND RESULTS

### Protocol development and optimization in *Nothofagus pumilio*

We initially focused on *Nothofagus pumilio* (Poepp. & Endl.) Krasser, a dominant tree species in the sub‐Antarctic temperate forests of Patagonia. This species belongs to the monotypic family Nothofagaceae within the order Fagales and is widely distributed from central Chile and the northern Patagonian Andes (35°S) to Tierra del Fuego (55°S) (Donoso, 1987; Veblen et al., 1996). Long‐read sequencing of *N. pumilio* is being conducted as part of the telomere‐to‐telomere (T2T) genome assembly project. For this purpose, obtaining pure HMW DNA is essential. However, the isolation of HMW DNA from non‐model plants growing in natural conditions, such as forest tree species, presents significant challenges due to the presence of secondary metabolites and other cellular interfering components, including compounds that hinder extraction efficiency and interfere with standard extraction methods (Aboul‐Maaty and Oraby, 2019; Jones et al., 2023).

To address this challenge in *N. pumilio*, we tested a wide range of extraction methods, including in‐house protocols and commercial kits. However, none of the approaches evaluated yielded DNA of sufficient quality or quantity from *N. pumilio* leaf tissue (Appendix S1; see Supporting Information with this article). Common issues included low DNA yield, recovery of fragmented DNA, suboptimal A_260_/A_280_ and A_260_/A_230_ ratios, and viscous DNA solutions that were difficult to handle. For instance, the protocol described by Healey et al. (2014) and the introduced modifications (Appendix S1) resulted in extremely viscous DNA solutions that were difficult to manipulate. Further purification attempts using column‐based methods resulted in significant loss of DNA and poor DNA quality. Similarly, magnetic bead–based methods, such as those described by Mayjonade et al. (2016), and commercial kits (e.g., NucleoSpin DNA extraction kit) failed to isolate DNA due to the jelly‐like consistencies of the extracts obtained after the addition of the lysis buffer, which prevented the magnetic beads from binding properly to the magnet or separating from the DNA.

We therefore optimized an in‐house DNA extraction method capable of yielding *N. pumilio* DNA with excellent quality and quantity for third‐generation sequencing. The key improvement was the use of cleaner starting material, which was achieved through the introduction of a preliminary nuclei purification step. Although it requires gram‐scale amounts of tissue, nuclei purification steps prior to DNA extraction have been proposed to significantly reduce cellular debris and secondary metabolites (Butto et al., 2023). This enhances the suitability of the extraction method for obtaining high‐quality DNA, particularly in biochemically challenging tissues. Once obtained, the nuclei‐enriched extract undergoes a CTAB‐based extraction followed by DNA purification using magnetic beads. The step‐by‐step protocol and needed reagents are presented in Appendix 1. This protocol does not require expensive purification kits and can be performed in any laboratory equipped with basic instrumentation, making it accessible for implementation in small laboratories worldwide. Additionally, it has proven to be efficient for the extraction of high‐quality and HMW DNA from leaves of all the tested species growing in natural conditions, as well as for leaves of the model plant *A. thaliana* growing in chambers. Below, we provide a brief description of the methodology and outline the critical steps necessary to ensure the protocol's success.

#### Preparation of the nuclei‐enriched extract

To adjust the nuclei purification process, we used 5 g of frozen tissue that was ground with a mortar and pestle. Liquid nitrogen was added continuously during grinding until a fine powder was obtained, keeping the material fully frozen, which allows the preservation of nuclei. In the case of *N. pumilio*, this initial grinding step lasted about 3–4 min; however, the grinding time depends on the hardness of the leaf tissue. Once fully pulverized, the tissue powder was resuspended at a concentration of 10% w/v (e.g., 5 g of fresh tissue per 50 mL buffer) in a MgSO₄‐based nuclei stabilization buffer pre‐chilled at 4°C (Yang et al., 2019). This buffer contains HEPES to stabilize pH and preserve nuclear integrity, Triton X‐100 (Dow Chemical Company, Midland, Michigan, USA) to facilitate cell lysis and nuclei purification, MgSO_4_ to stabilize chromatin, KCl to maintain ionic strength, and dithiothreitol (DTT) as a reducing agent (buffer component functions described according to Loureiro et al., 2021). All subsequent steps were carried out on ice to maintain a low temperature throughout the nuclei purification process and preserve nuclear integrity.

At this stage, it is essential to break up any clumps of material formed during tissue resuspension in the MgSO₄‐based nuclei stabilization buffer to facilitate the release of the nuclei into the solution. The mixture was incubated for 10 min with gentle agitation, followed by sequential filtration through progressively smaller pore sizes (340 µm, 120 µm, and 25 µm). This filtration sequence and set of pore sizes yielded the most effective results for nuclei purification. Starting filtration with pore sizes smaller than 340 µm (e.g., 120 µm) caused membrane clogging and required the application of strong pressure, often leading to nuclei rupture. Filtration was conducted using a custom‐built system—consisting of a plastic container with a perforated lid—specifically designed to eliminate the need for vacuum devices, making it suitable for laboratories with basic equipment and no access to vacuum systems (see Appendix S2A,B for the setup). The nuclei suspension is poured into the plastic container, after which the selected pore‐size filter is positioned over the container opening. The perforated lid is then closed, securing the filter at the top of the container (Appendix S2A). When the container is inverted, the solution passes through the filter by gravity, while leaf material and cell debris are retained. The filtrate is collected in a separate container placed below the device (Appendix S2B). If the nuclei suspension is viscous, as observed with *N. pumilio*, gentle tapping by hand on the base of the container can facilitate faster passage of the solution through the filter.

Following filtration, nuclei were separated from the residual tissue and cellular components by low‐speed centrifugation. Specifically, the filtrate was divided into 6‐mL aliquots, each placed in 15‐mL conical tubes, and centrifuged at 3220 × *g* for 12 min at 4°C. At this stage, nuclei should form a pellet, while the supernatant must be discarded. If the solution remains excessively dense and the pellet is poorly defined, as observed for the first centrifugation steps in *N. pumilio* (see Appendix S2C for visual reference), additional centrifugation cycles should be performed until the supernatant can be safely removed without disturbing the pellet. The resulting pellets were gently resuspended in 5 mL of fresh, cold, MgSO₄‐based nuclei stabilization buffer using a fine brush, followed by an additional centrifugation at 3000 × *g* for 10 min at 4°C. This washing procedure was repeated until the supernatant became completely clear. At this point, a distinct white layer in the pellet should be visible, confirming the presence of nuclei (Zhao et al., 2022) (see Appendix S2D,E for visual reference). After finishing the washing steps, the supernatant was discarded and 1 mL of fresh, cold nuclei purification buffer was added to each tube. The nuclei suspensions can be stored at −80°C until further DNA extraction.

#### DNA extraction from the nuclei extract

Following nuclei purification, DNA extraction was carried out using a CTAB‐based protocol. At this stage, proteinase K must be incorporated into the lysis buffer to maximize DNA yield. As shown in Appendix S3, the incorporation of proteinase K resulted in a 3.8‐fold increase in DNA yield compared to extractions performed without the enzyme, after resuspension in step 20 (Appendix 1). This improvement can be attributed to the proteolytic activity of proteinase K, which facilitates the removal of nucleases that degrade DNA and promotes the breakdown of histone proteins, thereby enhancing the release of nucleic acids (Varma et al., 2007).

Following lysis, the sample underwent two purification steps with chloroform:isoamyl alcohol to remove proteins and other cellular compounds. Given the importance of RNA removal for downstream sequencing, RNase A was added immediately after the first chloroform:isoamyl alcohol extraction step. DNA was subsequently precipitated using isopropanol, washed four times with 70% ethanol to remove residual salts and impurities, and finally resuspended in Tris‐EDTA (TE) buffer. All pipetting steps must be performed using wide‐bore tips to prevent DNA shearing. Following ethanol washes, DNA pellets can be pooled into a single tube prior to final resuspension, if desired.

We then evaluated two DNA purification strategies: one based on commercial silica column kits and another using magnetic bead technology. For the column‐based method, we adapted the Monarch Genomic DNA Purification Kit (catalog no. T3010S; New England Biolabs, Ipswich, Massachusetts, USA) due its proven efficacy in animal samples for DNA extraction and subsequent sequencing (New England Biolabs, 2020) and its relatively low cost (approximately 50% less than other commercial kits). Among the tested buffers, the blood lysis buffer was the only one that yielded high‐purity DNA. The second purification method tested was based on the use of magnetic beads. This method relies on the reversible binding of DNA to paramagnetic beads in the presence of polyethylene glycol (PEG) and salt. By adjusting PEG concentrations, DNA can be selectively retained while low‐molecular‐weight DNA and contaminants are efficiently removed. Furthermore, employing magnetic beads for DNA purification eliminates the need for centrifugation, thereby reducing mechanical stress on the DNA (Mayjonade et al., 2016). Although a magnetic rack is required to separate the beads, it can be easily fabricated using 3D printing and appropriate magnets (Appendix S2F), making this procedure highly accessible for small laboratories equipped with basic instrumentation.

For the magnetic bead purification procedure, up to 10 µg of DNA was processed per reaction. The DNA solution was adjusted to a final volume of 500 µL with TE buffer and mixed with an equal volume (500 µL) of pre‐prepared magnetic bead solution (see Appendix 1). The mixture was gently homogenized and incubated at room temperature for 10 min to facilitate DNA binding to the beads. Following incubation, the tube was placed on a magnetic rack until the beads had fully migrated and the solution became clear, and the supernatant was then carefully removed without disturbing the beads. The beads were then washed four times with 1 mL of 70% ethanol and air‐dried for 15 s before elution. Finally, DNA was eluted by resuspending the beads in TE buffer pre‐warmed to 50°C, followed by a 10‐min incubation at room temperature to facilitate DNA release. The tube was then placed on the magnetic rack, and the eluted DNA solution was carefully transferred to a new tube, avoiding bead carryover.

#### Evaluation of DNA quantity and quality

DNA quantity and quality were assessed at two critical points within the experimental workflow: after step 20 (following resuspension after the ethanol wash steps) and after step 32 (upon completion of nucleic acid recovery via column‐based or magnetic bead–based methodologies) (Appendix 1). The rationale for evaluating DNA at the intermediate step (i.e., resuspension after the ethanol washes) was to elucidate the effects of the last purification processes (i.e., column‐ and bead‐based clean‐up) on DNA integrity and purity, as well as to estimate potential nucleic acid loss. Quantification was performed using a Qubit Fluorometer (Thermo Fisher Scientific, Waltham, Massachusetts, USA), which employs fluorescence‐based detection to selectively measure double‐stranded DNA (dsDNA). Purity metrics were obtained using the NanoDrop 2000 spectrophotometer (Thermo Fisher Scientific) by analyzing the ratio of absorbance at 260 nm and 280 nm (A_260_/A_280_) and at 260 nm and 230 nm (A_260_/A_230_), as well as the NanoDrop/Qubit concentration ratios. The NanoDrop quantifies total UV‐absorbing molecules by measuring absorbance at 260 nm, whereas Qubit specifically detects dsDNA via fluorescence‐based methods (Versmessen et al., 2024). This distinction makes the NanoDrop/Qubit ratio a valuable quality control metric for sequencing platforms. In this regard, NanoDrop/Qubit ratios below 2 generally indicate high‐purity DNA and are considered suitable for downstream sequencing applications. Additionally, A_260_/A_280_ ratios of ~1.75–1.8 and A_260_/A_230_ ratios between 2.0 and 2.2 are commonly required by sequencing providers to ensure the quality of input DNA (Oxford Nanopore Technologies, 2017; Pacific Biosciences, 2021).

**Figure 1:**
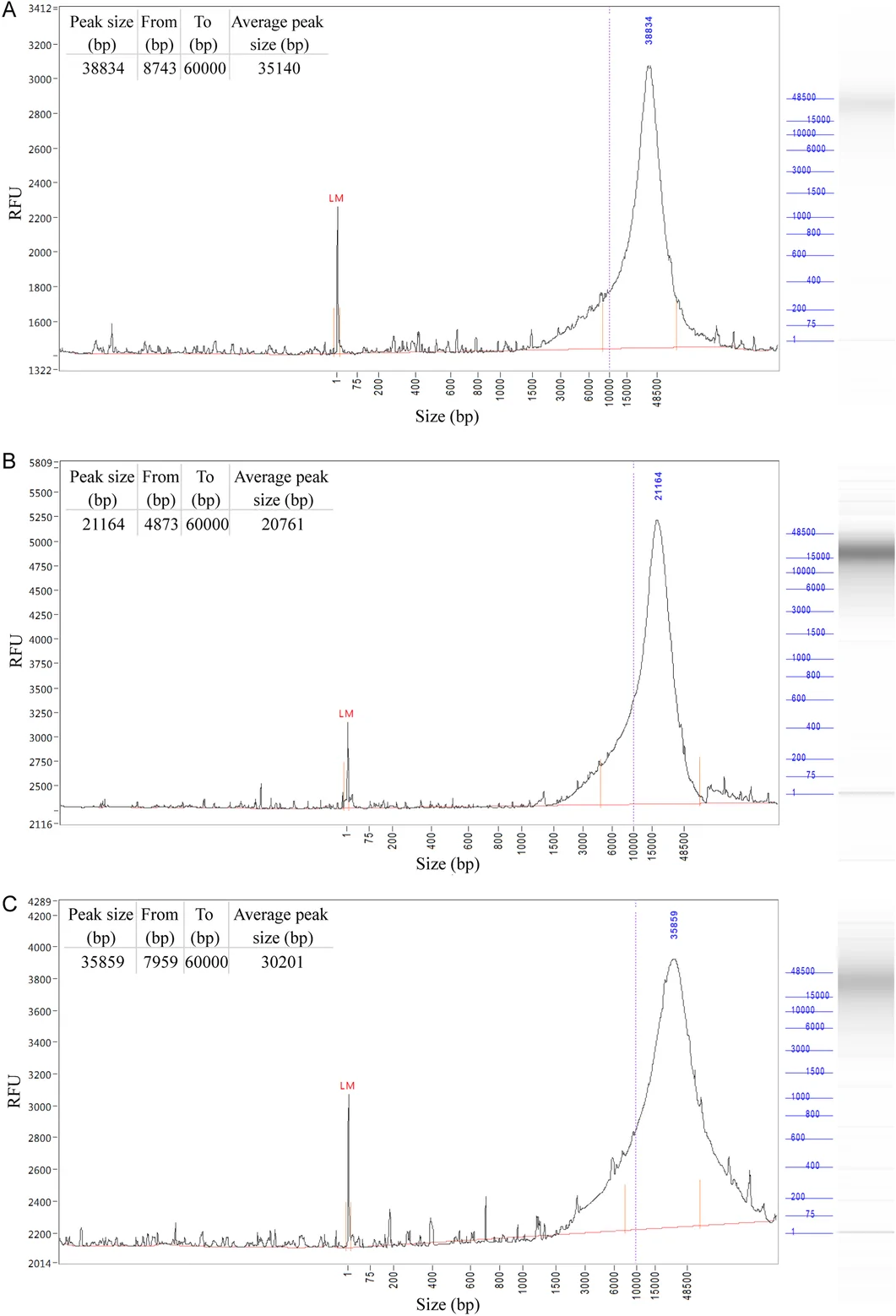
Size distribution profiles of *Nothofagus pumilio* genomic DNA analyzed with the Fragment Analyzer system. Each panel presents two complementary views: on the left, an electropherogram illustrates the relationship between relative fluorescence units (RFU) and fragment size in base pairs (bp); on the right, a capillary electrophoresis image depicts the physical DNA profile of the sample. (A) Genomic DNA before purification, (B) DNA purified using silica columns, and (C) DNA purified using magnetic beads. All runs included a lower marker (LM) to establish the minimum resolution threshold and a ladder for fragment size and concentration calibration. The main peaks represent the predominant fragment sizes. Red vertical lines delineate the size range used to calculate the average peak size. Samples were processed using the DNF‐468‐HS Genomic DNA 50 kb kit in the Fragment Analyzer 5200 system (Agilent Technologies) equipped with a 12‐capillary array according to the manufacturer's instructions, at the Genomics Unit of INTA (Castelar, Argentina). Data were acquired using FA software (version 1.1.0.11, Agilent Technologies) and data analysis was done with PROSize 3.0.1.6 software (https://prosize.software.informer.com/).

As shown in Table 1, both column‐based and magnetic bead–based methods yielded DNA with A_260_/A_280_ values over 1.8, A_260_/A_230_ values above 2.0, and NanoDrop/Qubit concentration ratios of approximately 1.1. For magnetic bead–based purification, capillary electrophoresis showed high integrity of the extracted DNA, revealing a peak fragment size of 35,859 bp. This size was close to the fragment size peak observed for *N. pumilio* DNA resuspended after ethanol washes (38,834 bp). In contrast, eluted DNA from column‐based methods exhibited a lower peak size (21,164 bp), indicating fragmentation or degradation during the final clean‐up step (Figure 1). Additionally, a significant advantage of magnetic bead–based purification is a roughly 70% reduction in the cost per purification reaction compared to the commercial column‐based method. Based on these results, we opted for the magnetic bead–based purification step as the last step of the protocol.

**Table 1:** Comparison of silica column–based and magnetic bead–based DNA purification methods for *Nothofagus pumilio* from 5 g of fresh tissue.

Species	Purification method	Treatment	DNA concentration (ng/µL) (NanoDrop)	DNA concentration (ng/µL) (Qubit)	NanoDrop/Qubit ratio	A_260_/A_280_ ratio	A_260_/A_230_ ratio	DNA yield (µg)	DNA yield/gram of starting material (µg/g)
*Nothofagus pumilio*	Silica columns	Before purification	1613.2	454.0	3.55	1.88	1.97	22.70	4.54
		Purified 1st elution	89.0	78.0	1.14	1.90	2.47	3.90	0.78
		Purified 2nd elution	76.5	70.4	1.09	1.88	2.66	3.52	0.70
	Magnetic beads	Before purification	957.6	864.0	1.11	1.73	1.34	43.20	8.64
		Purified	83.2	74.0	1.12	1.86	2.38	3.70	0.74

#### Optimizing the amount of input material

In order to optimize the amount of starting leaf material for facilitating the filtration and centrifugation steps during the preparation of nuclei‐enriched extracts for DNA extraction, we evaluated the performance of our protocol using 1 and 2 g of tissue and compared the results with those obtained using 5 g of tissue (Appendix S4). Although 5 g yielded the highest DNA recovery per gram of input, using 2 g was more practical, as it reduced working volumes, simplified filtrate handling, and shortened both the filtration and centrifugation steps, while still providing sufficient DNA for downstream applications. In contrast, extractions starting from 1 g of tissue resulted in low DNA yield, likely due to significant retention of a large proportion of the filtrate within the filter matrix. Accordingly, the standardized protocol described in Appendix 1 employs 2 g of starting material to achieve an optimal balance between DNA yield and ease of handling.

### Protocol robustness across diverse plant species

To assess the robustness of the DNA extraction method, we tested leaf material from a wide range of plant species, including both wild and model organisms. All tree species were sampled from natural environments, whereas *A. thaliana* (Col‐0) was cultivated under controlled indoor conditions. These plants were grown following the methodology outlined in Serrano et al. (2021) and maintained for 40 days in growth chambers (LT‐36VL; Percival Scientific, Perry, Iowa, USA) at 22°C under a long‐day photoperiod (16 h of light at 200 μmol·m^−^²·s^−^¹/8 h darkness) until leaf harvesting. The wild species comprised a diverse group of Patagonian trees. Among those, five *Nothofagus* species native to Argentina (*N. obliqua* (Mirb.) Oerst., *N. antarctica* (G. Forst.) Oerst., *N. alpina* (Poepp. ex A. DC.) Oerst., *N. dombeyi* (Mirb.) Oerst., and *N. betuloides* (Mirb.) Oerst.) and three native conifer species (*Fitzroya cupressoides* (Molina) I.M. Johnst., *Austrocedrus chilensis* (D. Don) Pic. Serm. & Bizzarri, and *Araucaria araucana* (Molina) K. Koch) were included. We also tested two commercially important conifers: *Pseudotsuga menziesii* (Mirb.) Franco and *Pinus ponderosa* P. Lawson & C. Lawson. Model species included *Populus trichocarpa* and the aforementioned *A. thaliana*. Detailed information on the collection sites, including mean annual temperature, precipitation, and altitude, are presented in Appendix S5. Overall, the method yielded consistent and reproducible results across all tested species and biological replicates, comparable in quality to those obtained for *N. pumilio*. Failures or inconsistencies were never observed throughout its application. This was reflected in A_260_/A_280_ ratios above 1.8, A_260_/A_230_ ratios above 2.0, NanoDrop/Qubit concentration ratios below 2, and high DNA integrity as revealed by capillary electrophoresis (Table 2).

**Table 2:** Performance of the optimized DNA extraction method across diverse tree species.^a^

Species	DNA concentration (ng/µL) (NanoDrop)	DNA concentration (ng/µL) (Qubit)	NanoDrop/Qubit ratio	A_260_/A_280_ ratio	A_260_/A_230_ ratio	DNA yield (µg)	Peak size (bp)	DNA yield/grams of starting material (µg/g)
*Nothofagus dombeyi*	98.0	74.8	1.31	1.87	2.30	3.74	42,653	1.87
*Nothofagus alpina*	141.4	112.0	1.26	1.85	2.19	5.60	36,175	2.80
*Nothofagus obliqua*	65.3	58.6	1.11	1.84	1.97	2.93	34,279	1.47
*Nothofagus antarctica*	112.3	112.4	1.00	1.84	2.33	5.62	49,130	2.81
*Nothofagus betuloides*	60.9	54.0	1.13	1.84	2.49	2.70	28,432	1.35
*Araucaria araucana*	106.9	99.0	1.08	1.82	2.49	4.95	52,438	2.48
*Fitzroya cupressoides*	77.6	67.6	1.15	1.82	2.32	3.38	43,444	1.69
*Austrocedrus chilensis*	104.0	98.2	1.06	1.82	2.22	4.91	40,283	2.46
*Pinus ponderosa*	72.9	65.0	1.12	1.81	2.27	3.25	41,834	1.63
*Pseudotsuga menziesii*	50.1	51.2	0.98	1.89	2.30	2.56	36,167	1.28
*Populus trichocarpa*	82.0	71.6	1.15	1.84	2.45	3.58	40,599	1.79
*Arabidopsis thaliana*	85.0	79.3	1.07	1.85	1.93	3.97	35,167	1.98

a Extractions were performed using 2 g of leaf material from different species, including *Nothofagus* species, gymnosperms, and the model species *Populus trichocarpa* and *Arabidopsis thaliana*.

### Testing downstream DNA sequencing with ONT and PacBio technologies

**Figure 2:**
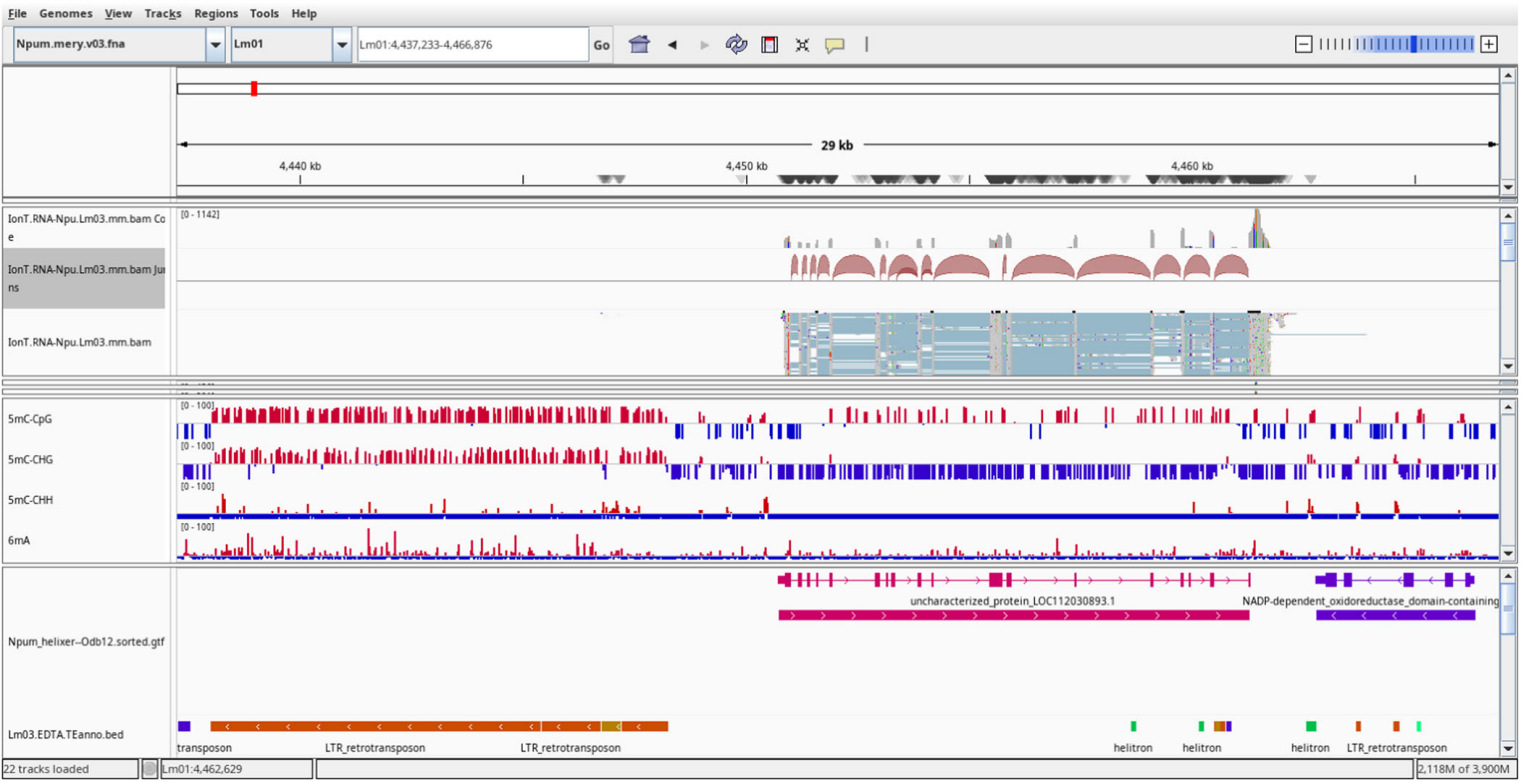
DNA methylation patterns in the context of transcriptional activity and genome structure visualized using the Integrative Genomics Viewer (IGV) (Robinson et al., 2011). From top to bottom, tracks depict: RNA‐Seq coverage as raw read depth, splicing junctions, and reads pileup (Ion Torrent, Thermo Fisher Scientific), total RNA sequencing was aligned with Minimap2 (Li, 2018); DNA methylation of 5mC in CpG, CHG, and CHH contexts and 6mA from ONT R10 sequencing, processed with Dorado (Oxford Nanopore Technologies, 2025); gene and transposable elements annotations, gene and untranslated region predictions via de novo gene coding with Helixer plant model (Holst et al., 2026); and coding and non‐coding transcripts inferred from total RNA‐Seq with Stringtie (Pertea et al., 2015) using Ion Torrent sequencing and transposable elements prediction with EDTA (Ou et al., 2019). This example shows a silenced transposon, which is heavily methylated, and a transcribed gene. The latter is unmethylated along the gene body in CHG and CHH contexts; however, in CpG context it is methylated differentially, which is consistent with the knowledge about relationships between gene expression and methylation in plants (Niederhuth and Schmitz, 2017).

To evaluate the suitability of the extracted DNA for distinct long‐read sequencing applications, we employed two third‐generation sequencing platforms: ONT, for direct DNA sequencing aimed at detecting base modifications, and PacBio HiFi sequencing (Pacific Biosciences, Menlo Park, California, USA), for genome assembly. ONT sequencing was performed on a PromethION 2 Solo device using an R10.4.1 flow cell (FLO‐PRO114M), with library preparation carried out using the Ligation Sequencing Kit V14 (SQK‐LSK114), following the manufacturer's instructions (https://nanoporetech.com; Oxford Nanopore Technologies, Oxford, United Kingdom). DNA samples obtained after step 20 (resuspended following ethanol washes) failed to yield sequence data, highlighting the necessity of high‐purity DNA for ONT workflows. In contrast, samples obtained from column or bead eluates at the final step of the described protocol enabled successful sequencing. Of these samples, DNA eluted from magnetic beads yielded longer ONT reads (N50 = 15.23 kbp) compared to DNA eluted from columns (N50 = 11 kbp), which is consistent with the recovery of higher‐molecular‐weight DNA using the bead‐based method (Figure 1), as described above. ONT runs yielded 25.76 Gbp (in a 72‐h run), with 2.8 Gbp sequenced during the first 3 h. The resulting ONT reads were suitable for detecting methylation in adenines (*N* ^6^‐methyladenine [6 mA]) and cytosines (5‐methylcytosine [5mC]) in CpG, CHG, and CHH contexts (Figure 2). For PacBio HiFi sequencing, DNA obtained from *Nothofagus obliqua* (*n* = 13; Ono, 1977) by the final adjusted method (Appendix 1) produced 38 Gbp of high‐quality long reads, with an N50 of 19.5 kbp. Assembly using Hifiasm (Cheng et al., 2021) with default parameters and DeepConsensus polishing (Baid et al., 2023) yielded a genome assembly of 549 Mbp, aligning closely with in silico genome size estimation (~700 Mbp). The genome assembly's N80 included the 13 larger contigs, with the largest contig spanning 60 Mbp in size, likely representing a full chromosome. Collectively, these results validate the robustness and versatility of the DNA extraction method described here, supporting its application across a range of long‐read sequencing tasks, from epigenetic profiling to de novo genome assembly.

## CONCLUSIONS

This study demonstrates the development of an effective, accessible protocol for isolating high‐purity and high‐molecular‐weight DNA from a wide range of non‐model and model plant species, including mature trees growing under natural conditions. By incorporating a nuclei purification step prior to CTAB extraction and magnetic bead–based clean‐up, the method consistently yielded DNA of high purity and fragment size suitable for third‐generation sequencing platforms such as ONT and PacBio. The approach overcame common limitations associated with endogenous plant components that often interfere with extraction and downstream applications, providing a cost‐effective alternative to commercial kits without compromising performance. Validation across multiple species confirmed the robustness and reproducibility of the protocol. This work provides a valuable tool for researchers aiming to generate long‐read genomic data from challenging plant materials, thereby facilitating comprehensive genome assemblies and epigenomic analyses in underrepresented taxa.

## AUTHOR CONTRIBUTIONS

M.V.A. coordinated the project. S.G. wrote the manuscript with help from M.V.A. C.F.M., E.P., F.A.R., D.W., and N.B. critically revised the manuscript. M.V.A. and C.F.M. helped S.G. in the preliminary nuclei purification trials. S.G. adjusted the final extraction protocol, acquired the DNA data, and performed DNA quantity and quality analysis. E.P. and S.G. prepared the ONT libraries, and E.P. carried out the ONT runs. F.A.R. carried out the PacBio sequencing, and F.A.R. and D.W. analyzed the data. N.B. performed ONT bioinformatics analysis. All authors approved the final version of the manuscript.

## Supporting information


**Appendix S1.** Results of commercial and published DNA extraction protocols applied to *Nothofagus pumilio*.

**Appendix S2.** Illustrative images depicting custom‐made tools and key stages in the extraction of enriched nuclear material.

**Appendix S3.** Effect of proteinase K addition during lysis on DNA yield and extraction efficiency in *Nothofagus pumilio*, after ethanol washes.

**Appendix S4.** DNA yield, purity, and extraction efficiency from different amounts of *Nothofagus pumilio* leaf material using the optimized extraction method.

**Appendix S5.** Locations and environmental data for sampled individuals of each species.

## Data Availability

The data supporting the findings of this study are available within the article text and its Supporting Information.

## Appendix 1 Detailed protocol for DNA extraction including the preliminary nuclei purification step.

### Equipment and supplies:

- Conical tubes (15 mL)
- Tubes (1.5 mL)
- Filters (120 and 340 μm)
- Miracloth (catalog no. 475855‐1R; Sigma‐Aldrich, St. Louis, Missouri, USA)
- Pipettes (P20, P200, P1000, P5000)
- Pipette tips (10 μL, 200 μL, 1000 μL, 5000 μL)
- Mortar and pestle
- Beaker
- Paintbrushes
- Swinging bucket centrifuge for 15‐mL conical tubes
- Fume hood
- Water bath
- Magnetic rack
- Liquid nitrogen
- Ice

### Reagents:

- Dithiothreitol (DTT)
- β‐mercaptoethanol (β‐ME)
- Ethylenediaminetetraacetic acid (EDTA)
- Tris(hydroxymethyl)aminomethane (Tris‐HCl)
- Sodium chloride (NaCl)
- Magnesium sulfate (MgSO_4_·7H_2_O)
- Potassium chloride (KCl)
- Polyethylene glycol 8000 (PEG)
- 4‐(2‐hydroxyethyl)piperazine‐1‐ethanesulfonic acid (HEPES)
- Triton X‐100 (Dow Chemical Company, Midland, Michigan, USA)
- Cetyltrimethylammonium bromide (CTAB)
- Polyvinylpyrrolidone (PVP) (viscosity‐average molecular weight [*M*_*v*_] = 40,000)
- Chloroform:isoamyl alcohol (24:1)
- Ethanol 70%
- Proteinase K
- RNase A
- Sera‐Mag carboxylate‐modified (E3) magnetic particles, 1 μm (catalog no. 44152105050250; Cytiva, Marlborough, Massachusetts, USA)
- double‐distilled H_2_O (ddH_2_O)

### Buffers and solutions:

**MgSO**_**4**_ **buffer** (500 mL):

1.23 g MgSO_4_·7H_2_O

1.85 g KCl

0.6 g HEPES

Dissolve in 480 mL of ddH_2_O, adjust pH to 8.0 using potassium hydroxide, and then bring to a final volume of 500 mL with ddH_2_O. Store at 4°C.

**MgSO**_**4**_ **‐based nuclei stabilization buffer** (100 mL)*:

96.5 mL MgSO_4_ buffer (pH 8.0)

0.5% v/v Triton X‐100

1 mL DTT (1 M)**

Bring to the final volume of 100 mL with ddH_2_O. Keep on ice after preparation.

*Prepare immediately before use.

**Add DTT immediately before use; DTT oxidizes rapidly in air.

**CTAB buffer:**

100 mM Tris‐HCl (pH 8)

2.5 mM EDTA (pH 8)

1.5 M NaCl

2% CTAB (w/v)

1% PVP‐40

0.3% β‐ME (add prior to use in a fume hood)

**TE buffer 1X:**

10 mM Tris‐HCl

1 mM EDTA

**PEG solution** in ddH_2_O:

18% PEG 8000

1 M NaCl

10 mM Tris‐HCl

1 mM EDTA

**Magnetic bead solution** (store at 4°C):

49 mL PEG solution in ddH_2_O

1 mL of washed magnetic beads (Sera‐Mag carboxylate‐modified [E3] magnetic particles; see under Reagents) resuspended in 1X TE buffer.

Instructions for the magnetic bead wash and resuspension in 1X TE:

Wash the beads four times with 1X TE buffer. To do this, pipette the desired volume of the commercial bead suspension into a tube and place it on a magnetic rack. Once the beads have migrated to the magnet, carefully discard the supernatant. Add the same volume of 1X TE buffer as the initial bead suspension (e.g., if 1 mL of commercial beads was used, add 1 mL of 1X TE buffer) and gently invert the tube 10 times to resuspend the beads. Place the tube back on the magnetic rack and repeat this washing step three additional times. After the beads are washed, resuspend them in 1X TE buffer and add them to the magnetic bead solution to reach a final concentration of 2% (e.g., mix 1 mL of resuspended beads with 49 mL of PEG solution to obtain 50 mL of magnetic bead solution).

### DNA extraction protocol:

1. Grind ~2 g of the frozen tissue (tissue weight:MgSO_4_‐based nuclei stabilization buffer ratio = 1:10) into fine powder in the presence of liquid nitrogen with a mortar and pestle, and then transfer the fine powder into an ice‐cold 500‐mL beaker containing ~20 mL of the MgSO_4_‐based nuclei stabilization buffer. The tissue must be mixed with the buffer very quickly; if the frozen tissue clumps, break it apart with a spatula or glass rod.
2. Gently mix the contents of the beaker on ice for 10 min using an orbital shaker at 40–60 rpm (1.9 cm orbit diameter) or by gentle manual agitation mimicking this motion.
3. Filter the mixture through a 340‐µm filter. Collect the filtrate in a beaker placed on ice. You can gently squeeze the filter to collect the remaining solution in the filter.
4. Filter the recovered extract through a 120‐µm filter. Collect the filtrate in a beaker placed on ice. You can gently squeeze the filter to collect the remaining solution in the filter.
5. Filter the recovered extract through a single layer of Miracloth (22–25 µm). Collect the filtrate in a beaker placed on ice.
6. Aliquot the filtrate (typically 10–18 mL total) into 15‐mL conical tubes, adding 6 mL per tube.
7. Pellet the homogenate at 3220 × *g* for 12 min at 4°C, then discard the supernatant. If the homogenate does not pellet, repeat this step until it does.
8. Discard the supernatant; add 5 mL of ice‐cold MgSO_4_‐based nuclei stabilization buffer to each conical tube and resuspend the pellet gently using a small paintbrush.
9. Pellet the homogenate by centrifugation at 3000 × *g* for 10 min at 4°C.
10. Repeat steps 8 to 9 until the suspension becomes clear.
11. Discard the supernatant; add 1 mL of ice‐cold MgSO_4_‐based nuclei stabilization buffer and resuspend the pellet gently using a small paintbrush.
12. If desired, pool the nuclei solution into a single tube.
  Note: At this point, the nuclei extract can be stored at −80°C to continue with the CTAB‐based DNA extraction the following day or at a later time.
13. Pour 3 mL of CTAB buffer (preheated at 65°C) containing 0.5% β‐ME into each nuclei extract tube. Add 20 µL of proteinase K. Incubate for 30 min at 50°C. During incubation, invert the tube gently every 10 min.
14. Add 1 volume of chloroform:isoamyl alcohol (24:1). Gently invert the tube 20 times. Centrifuge at 2400 × *g* for 10 min at 4°C.
15. Transfer the aqueous phase into a new tube. Add 10 µL of RNase A (10 mg/mL) and incubate for 30 min at 37°C.
16. Add 1 volume of chloroform:isoamyl alcohol (24:1). Gently invert the tube 20 times. Centrifuge at 2400 × *g* for 10 min at 4°C.
17. Transfer the aqueous phase into a new tube. Add 1 volume of isopropanol, invert the tube gently, and incubate for 30 min at 4°C. A silk‐like DNA precipitate should become visible. Once the silk‐like DNA is visible in the solution, stop inverting the tube, as excessive mixing may cause genomic DNA to aggregate and become too dense to dissolve.
18. Centrifuge at 2400 × *g* for 10 min at 4°C.
19. Discard the supernatant. Wash 3–4 times with 1 mL of 70% ethanol.
20. Remove the residual ethanol by centrifugation for 5 s at maximum speed and aspirating it with a pipette from the bottom of the tube, without disturbing the pellet. Air‐dry the DNA and resuspend in 50 µL of 1X TE.
21. For each purification reaction, take up to 10 µg of DNA and adjust the DNA solution to a final volume of 500 µL with 1X TE buffer.
22. Add 500 µL of magnetic bead solution that has been previously equilibrated to room temperature.
23. Mix gently and incubate for 10 min at room temperature.
24. Place the tube on a magnetic rack.
25. Once the magnetic beads are fully attracted to the magnet and the solution appears clear, carefully remove the supernatant without disturbing the bead pellet.
26. Remove the tube from the magnetic rack and add 1 mL of 70% ethanol.
27. Place the tube back on the magnetic rack and, once the beads have migrated to the magnet, carefully discard the ethanol.
28. Repeat steps 26 and 27 three times.
29. Briefly centrifuge the tube, place it on the magnetic rack, and remove any residual ethanol.
30. Air‐dry the pellet for 15 s.
31. Add 50 µL of 1X TE buffer preheated at 50°C. Incubate for 10 min at room temperature to promote DNA elution.
32. Place the tube on the magnetic rack and allow the beads to fully migrate to the magnet. Using a wide‐bore pipette tip, carefully transfer the solution containing the extracted DNA to a new tube.

Note: Magnetic beads may require additional time to migrate during the final elution step. If necessary, leave the tube on the magnetic rack at 4°C overnight to ensure complete bead separation.
