# Supplementary material for: A cost‐effective DNA extraction protocol for long‐read sequencing in non‐model plants
Source: Appl Plant Sci. 2026 May 7;14(4):e70055. doi: 10.1002/aps3.70055 (PMC13495970; doi:10.1002/aps3.70055)
Supplement: Supplementary file 1 [file APS3-14-e70055-s004.pdf]

DOI 10.1002/aps3.70055

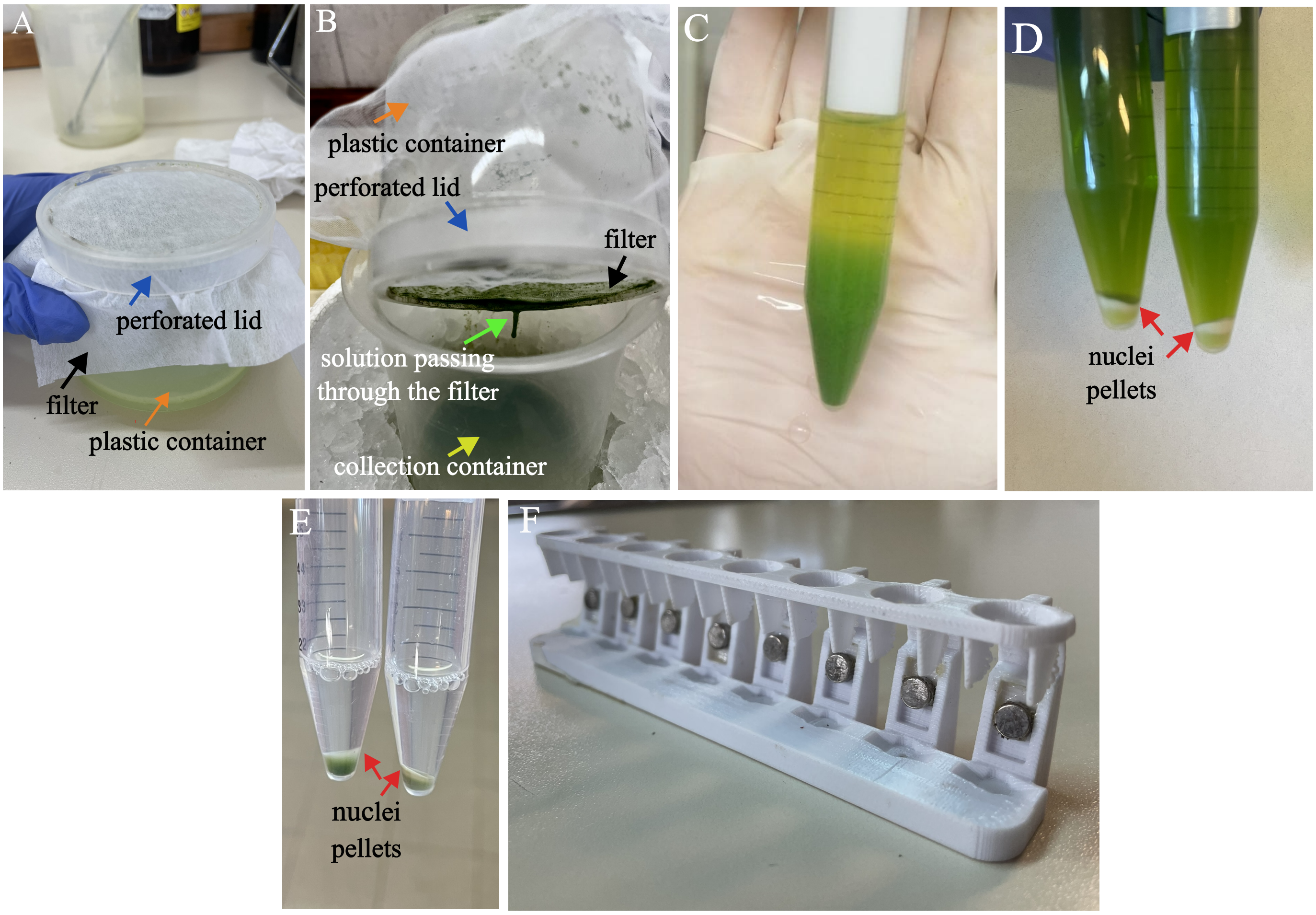

Appendix S2. Illustrative images depicting custom-made tools and key stages in the extraction of enriched nuclear material. (A, B) Custom-designed filtering device used during nuclei isolation from leaf tissues (steps 3–5 of the described methodology; see Appendix 1). The orange arrow shows the plastic container where the nuclei suspension is poured, the blue arrows indicate the perforated lid that holds the filter, the black arrow indicates the filter, the green arrow points to the solution passing through the filter, and the yellow arrow indicates the collecting container. (C) Image providing a visual reference of the poorly defined pellets observed in species where the nuclei extract becomes overly dense, as mentioned in step 7 of Appendix 1. (D, E) Images showing white nuclei during (D) and after (E) the centrifugation process (steps 7–10 of the described methodology; see Appendix 1). Red arrows mark the location of the nuclei pellet. (F) Custom-built magnetic rack employed for DNA purification using magnetic bead technology.
