# Supplementary material for: A cost‐effective DNA extraction protocol for long‐read sequencing in non‐model plants
Source: Appl Plant Sci. 2026 May 7;14(4):e70055. doi: 10.1002/aps3.70055 (PMC13495970; doi:10.1002/aps3.70055)
Supplement: Supplementary file 2 [file APS3-14-e70055-s001.docx]

**Appendix S4.** DNA yield, purity, and extraction efficiency from different amounts of *Nothofagus pumilio* leaf material using the optimized extraction method. DNA extraction was performed using 5 g, 2 g, and 1 g of fresh leaves. The table shows DNA concentration measured by NanoDrop and Qubit (ng/µL), NanoDrop/Qubit ratios, purity (A_260_/A_280_ and A_260_/A_230_ ratios), DNA yield (µg), and the extraction efficiency (µg per gram of starting material).

| **Species** | **Amount of starting  material (g)** | **Treatment** | **DNA concentration ng/µL (NanoDrop)** | **DNA concentration ng/µL  (Qubit)** | **NanoDrop/Qubit ratio** | **A_260_/A_280_ ratio** | **A_260_/A_230_ ratio** | **DNA yield (µg)** | **DNA yield/grams of starting material (µg/g)** |
| --- | --- | --- | --- | --- | --- | --- | --- | --- | --- |
| *Nothofagus  pumilio* | 5 | before purification | 957.6 | 864.0 | 1.11 | 1.73 | 1.34 | 86.40 | 5.92 |
|  |  | purified | 83.2 | 74.0 | 1.12 | 1.86 | 2.38 | 29.60 |  |
|  | 2 | before purification | 457.7 | 160.2 | 2.86 | 1.84 | 1.74 | 8.01 | 3.90 |
|  |  | purified | 240.4 | 156.0 | 1.54 | 1.79 | 1.83 | 7.80 |  |
|  | 1 | before purification | 49.3 | 29.6 | 1.67 | 1.87 | 1.95 | 1.48 | 0.35 |
|  |  | purified | 12.6 | 7.0 | 1.80 | 1.76 | 1.66 | 0.35 |  |
