# Supplementary material for: A cost‐effective DNA extraction protocol for long‐read sequencing in non‐model plants
Source: Appl Plant Sci. 2026 May 7;14(4):e70055. doi: 10.1002/aps3.70055 (PMC13495970; doi:10.1002/aps3.70055)
Supplement: Supplementary file 3 [file APS3-14-e70055-s003.docx]

**Appendix S3.** Effect of proteinase K addition during lysis on DNA yield and extraction efficiency in *Nothofagus pumilio*, after ethanol washes. Extractions were performed using the optimized method, with or without the addition of proteinase K during the lysis step. The table shows DNA concentration measured by NanoDrop and Qubit (ng/µL), NanoDrop/Qubit ratios, purity (A_260_/A_280_ and A_260_/A_230_ ratios), DNA yield (µg), and the extraction efficiency (µg per gram of starting material).

| **Species** | **Treatment** | **DNA concentration (ng/µL) (NanoDrop)** | **DNA concentration (ng/µL)**  **(Qubit)** | **NanoDrop/Qubit ratio** | **A_260_/A_280_ ratio** | **A_260_/A_230_ ratio** | **DNA yield (µg)** | **DNA yield/grams of starting material (µg/g)** |
| --- | --- | --- | --- | --- | --- | --- | --- | --- |
| *Nothofagus pumilio* | without proteinase K | 1613.2 | 454.0 | 3.55 | 1.91 | 1.87 | 22.70 | 4.54 |
|  | with proteinase K | 957.6 | 864.0 | 1.11 | 1.73 | 1.34 | 86.40 | 17.28 |
