# Supplementary material for: A cost‐effective DNA extraction protocol for long‐read sequencing in non‐model plants
Source: Appl Plant Sci. 2026 May 7;14(4):e70055. doi: 10.1002/aps3.70055 (PMC13495970; doi:10.1002/aps3.70055)
Supplement: Supplementary file 4 [file APS3-14-e70055-s005.docx]

**Appendix S5.** Locations and environmental data for sampled individuals of each species.

| **Species** | **Location of sampled  individuals** | **Mean annual  precipitation (mm)** | **Altitude (m a.s.l)** |
| --- | --- | --- | --- |
| *Nothofagus pumilio* | 41°15'24"S 71°16'53"W | 800–900 | 1313 |
| *Nothofagus dombeyi* | 41°10'41"S 71°24'53"W | 900–1000 | 832 |
| *Nothofagus alpina* | 41°07'23"S 71°14'57"W | 700–800 | 791 |
| *Nothofagus obliqua* | 41°07'25''S 71°15'01''W | 700–800 | 787 |
| *Nothofagus antartica* | 41°12'33"S 71°18'46"W | 800–900 | 981 |
| *Nothofagus betuloides* | 43°07'24"S 71°33'41"W | 700–800 | 378 |
| *Araucaria araucana* | 41°07'26''S 71°15'01''W | 700–800 | 787 |
| *Fitzroya cupressoides* | 41°22'31"S 71°45'04"W | 1800–2000 | 725 |
| *Austrocedrus chilensis* | 41°07'23"S 71°14'57"W | 700–800 | 791 |
| *Pinus ponderosa* | 41°07'26"S 71°15'06"W | 700–800 | 786 |
| *Pseudotsuga menziesii* | 41°07'26"S 71°15'06"W | 700–800 | 786 |
| *Populus trichocarpa* | 41°07'24''S 71°14'57''W | 700–800 | 790 |
